# Integrated Multi‐Omics Analysis of Human Cerebrospinal Fluid in Traumatic Spinal Cord Injury: An Exploratory Pilot Study

**DOI:** 10.1002/cns.71242

**Published:** 2026-11-02

**Authors:** Wenkang Jiang, Yao Wang, Wei Miao, Huandong Liu, Qiujiang Qiao, Ting Jiang, Chenyan Shen, Zetian Zhou, Huilin Cheng, Ning Li

**Affiliations:** ^1^ School of Medicine Southeast University Nanjing China; ^2^ Department of Neurosurgery, Huashan Hospital Fudan University Shanghai China; ^3^ Department of Neurosurgery, Zhongda Hospital Southeast University Nanjing China; ^4^ Department of Neurosurgery, Taikang Xianlin Drum Tower Hospital, Affiliated Hospital of Medical School Nanjing University Nanjing China; ^5^ College of Future Technology The Hong Kong University of Science and Technology (Guangzhou) Guangzhou China

**Keywords:** amino acid metabolism, cerebrospinal fluid, GOT2, metabolomics, proteomics, traumatic spinal cord injury

## Abstract

**Objective:**

Acute traumatic spinal cord injury (TSCI) is a devastating neurological disorder with limited therapeutic options. We aimed to characterize the human cerebrospinal fluid (CSF) microenvironment to identify novel multi‐omics signatures and potential mechanistic hypotheses.

**Methods:**

CSF from 13 acute cervical TSCI patients and 13 controls was prospectively collected. A discovery cohort (*n* = 10) underwent untargeted proteomics and metabolomics. Following differential and functional enrichment analyses, sequential multi‐omics integration was applied to extract cross‐omics features. Prioritized candidates were subsequently validated via ELISA and targeted metabolomics in an independent validation cohort (*n* = 16).

**Results:**

We identified 216 differentially expressed proteins and 26 metabolites, highlighting extensive extracellular matrix remodeling alongside profound purine and amino acid metabolism dysregulation. Integrated analyses converged on downregulated mitochondrial aspartate aminotransferase (GOT2) as a candidate marker. Targeted correlation analysis revealed that GOT2 selectively associated with specific co‐mapped metabolites that were concurrently downregulated, notably methionine and 4‐acetamidobutanoic acid. The GOT2‐associated metabolic signature was experimentally validated in the independent cohort.

**Conclusions:**

This exploratory multi‐omics study unveils the acute human TSCI molecular landscape, nominating a novel GOT2‐associated metabolic signature that warrants further mechanistic investigation regarding its pathogenic implications and potential therapeutic relevance.

## Graphical abstract

Integrated proteomic and metabolomic profiling of human cerebrospinal fluid outlines a distinct molecular landscape of acute traumatic spinal cord injury. This exploratory analysis highlights a novel GOT2‐associated metabolic signature, involving concurrent decreases of GOT2, methionine, and 4‐acetamidobutanoic acid. These findings warrant further mechanistic investigation.

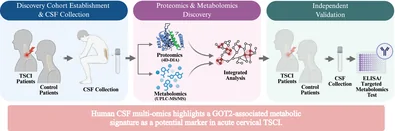

## 1 Introduction

Traumatic spinal cord injury (TSCI) remains a devastating neurological disorder causing permanent neurologic disability [1]. As a leading contributor to global long‐term disability [2], TSCI imposes profound economic and psychosocial burdens on society due to lifelong care requirements and loss of productivity [3]. Despite advances in acute care, neurosurgery, and rehabilitation, functional recovery remains limited [4]. To date, no therapies can effectively achieve meaningful neurological recovery [5], underscoring an urgent need for neuroprotective and regenerative strategies.

The pathogenic cascade of acute TSCI unfolds in two phases [6]. The primary mechanical insult is irreversible and is immediately followed by a secondary injury cascade. The secondary phase unleashes a host of deleterious processes, including inflammation, ischemia, excitotoxicity, and oxidative stress, which collectively expand the lesion and impede neural repair [7]. However, their molecular underpinnings remain incompletely understood, hindering the development of effective treatments [8].

High‐throughput omics technologies, such as proteomics and metabolomics, allow for systematic profiling of biological changes [9, 10]. While proteomics provides a snapshot of the cellular machinery, metabolomics captures the downstream phenotypic activity. Integrating these layers offers deeper mechanistic insights into perturbed signaling pathways [11, 12]. To date, most TSCI research has relied on animal models or single‐omics analysis [13, 14], which may not fully capture the human conditions. Specifically, integrated proteomic and metabolomic investigations of human cerebrospinal fluid (CSF) in acute TSCI are lacking.

To bridge this critical gap, we performed the first integrated proteomic and metabolomic analysis of CSF from patients with acute cervical TSCI, the most common type of spinal injury [15]. Using state‐of‐the‐art 4D label‐free data‐independent acquisition (DIA) proteomics and untargeted high‐resolution mass spectrometry metabolomics, we aimed to: (1) comprehensively characterize the proteomic and metabolomic signatures of the acute phase of human cervical TSCI; (2) identify key perturbed biological processes through multi‐omics integration; and (3) explore potential mechanistic hypotheses.

## 2 Methods

### 2.1 Study Design and Patient Enrollment

A total of 26 participants were enrolled between February 2023 and September 2025 at the Department of Neurosurgery, Zhongda Hospital, 13 in the spinal cord injury group (SCI group), and 13 in the control group (CON group). The cohort was randomly divided into a discovery cohort (*n* = 5 per group) for untargeted omics, and an independent validation cohort (*n* = 8 per group) for targeted biochemical verification.

The inclusion criteria for the SCI group were: (1) age 18–80; (2) traumatic etiology with cervical TSCI confirmed by MRI/CT; (3) American Spinal Injury Association Impairment Scale (AIS) grade A or B; (4) undergoing emergency decompression surgery within 48 h; and (5) intact dura mater confirmed intraoperatively. The exclusion criteria were: (1) non‐traumatic SCI; (2) concurrent severe trauma to other organ systems; (3) contraindications to lumbar puncture; (4) intraoperative dural violation; and (5) comorbidities including endocrine disorders, autoimmune or rheumatological diseases.

The CON group consisted of iNPH patients with no history of trauma or other central nervous system (CNS) disorders. To minimize background noise, any cases with abnormal CSF laboratory indices (e.g., cell counts, glucose, or protein levels) were strictly excluded for the CON group.

### 2.2 CSF Collection and Processing

For TSCI patients, approximately 4 mL of CSF was collected via lumbar puncture immediately following emergency decompression surgery under general anesthesia. For control patients, an equivalent volume was collected during routine diagnostic lumbar puncture. Half of each sample was sent to the Clinical Laboratory of Zhongda Hospital for CSF routine and biochemical tests, and the remaining half was centrifuged (3000 rpm, 15 min, 4°C), aliquoted, and stored at −80°C for further analysis.

### 2.3 4D Label‐Free DIA Proteomics

CSF samples (100 μL) were lysed in buffer containing protease inhibitors (Beyotime, China) and urea. After centrifugation (15,000 rpm, 4°C, 10 min), protein concentration was determined via BCA assay (Thermo Fisher Scientific, USA) and adjusted to 1 μg/μL. Proteins (100 μL) were reduced with 10 mM dithiothreitol (56°C, 1 h; Sangon Biotech, China), alkylated with 25 mM iodoacetamide (dark, 45 min; Sangon Biotech), and digested using the filter‐aided sample preparation (FASP) method at 37°C overnight [16]. Peptides were centrifuged (14,000 rpm, 10 min, 4°C), acidified, desalted with C18 cartridges, dried under vacuum, and reconstituted in 0.1% formic acid (Thermo Fisher Scientific).

Peptide separation was performed on a NanoElute2 system (Bruker, Germany) with an Aurora Ultimate CSI C18 column (75 μm × 25 cm, 1.7 μm). Mobile phase A was water with 0.1% formic acid, and mobile phase B was acetonitrile with 0.1% formic acid. A 60‐min gradient was used at 300 nL/min. Peptides were analyzed using a TimsTOF HT mass spectrometer (Bruker, Germany) in positive ion mode with DIA and parallel accumulation–serial fragmentation (PASEF). The m/z range was 300–1500, with an isolation window of 25.5 Da (12.27–122.81 Da). Ion source voltage was 1.6 kV, and ion mobility ranged from 0.6–1.4 V·s/cm^2^. Data acquisition was controlled by Bruker Compass Hystar (version 6.2).

DIA data were processed using Spectronaut (version 18.0) against the UniProt Human Proteome database. Search parameters included: Trypsin/P specificity, ≤ 2 missed cleavages, minimum peptide length of 6 amino acids, ≤ 3 modifications per peptide, mass tolerances of 10 ppm (precursor) and 0.02 Da (fragment). Fixed modification was carbamidomethyl (C); variable modifications included oxidation (M) and acetyl (N‐terminal). False discovery rate (FDR) was < 1.0% at peptide and protein levels. Proteins required ≥ 1 unique peptide for identification. Default settings were applied for other parameters.

### 2.4 Untargeted High‐Resolution Mass Spectrometry Metabolomics

Metabolites were extracted from 100 μL CSF using 500 μL acetonitrile (Thermo Fisher Scientific). Following protein precipitation (−20°C, 2 h) and centrifugation (20,000 rpm, 4°C, 10 min), supernatants were vacuum‐dried and reconstituted in 100 μL acetonitrile/water (3:1, v/v). A Quality Control (QC) sample was generated by pooling 10 μL aliquots from all samples.

Chromatographic separation was performed on a Thermo Vanquish ultra‐performance liquid chromatography (UPLC) system (Thermo Fisher Scientific, USA) with an ACQUITY UPLC BEH C18 column (100 mm × 2.1 mm, 1.8 μm; Waters, UK) at 40°C and a flow rate of 0.35 mL/min. Mobile phases were solvent A (ultrapure water with 5 mmol/L ammonium acetate and 5 mmol/L acetic acid; Thermo Fisher Scientific, USA) and solvent B (LC–MS grade acetonitrile; Thermo Fisher Scientific, USA). Injection volume was 4 μL. Mass spectrometry was conducted on an Orbitrap Exploris 120 (Thermo Fisher Scientific, Germany) in positive and negative ionization modes (spray voltages: +3.8 kV, −3.2 kV). Data were acquired in data‐dependent acquisition (DDA) mode (mass range: 70–1050 Da, resolution: 70,000 at m/z 200, AGC target: Standard, maximum injection time: 100 ms). MS2 scans used 17,500 resolution with dynamic exclusion, selecting the top 45 precursor ions (intensity threshold: 5000).

Raw data were converted to mzXML using MSConvert (Proteowizard, version 3.0). Chromatographic peaks were detected with XCMS (version 3.24.0), extracting m/z, retention time, and peak area. Metabolites were putatively identified at Metabolomics Standards Initiative (MSI) Level 2 using a tiered approach: MS1 via exact mass matching against HMDB and KEGG databases using metaX (version 1.4.16), and MS2 via tandem mass spectrometry (MS/MS) spectral matching against the standard HMDB and KEGG reference libraries to resolve isomer ambiguity.

### 2.5 Bioinformatic Analysis

All bioinformatic workflows were executed using R (v4.3.1). Candidate differentially expressed proteins (DEPs) were selected through fold‐change (FC) > 1.5 or < 0.67 and nominal *t*‐test *p*‐value < 0.05 [10]. Candidate differentially expressed metabolites (DEMs) were selected through FC > 1.5 or < 0.67, nominal *t*‐test *p*‐value < 0.05, and a Variable Importance in Projection (VIP) score > 1 derived from an Orthogonal Partial Least Squares‐Discriminant Analysis (OPLS‐DA) model [10]. Features meeting these criteria are operationally referred to as candidate DEPs and DEMs throughout this exploratory study.

Functional enrichment was then performed. DEPs were mapped to Gene Ontology (GO) and KEGG databases, while DEMs were analyzed against the KEGG database. Pathways with a hypergeometric *p*‐value < 0.05 were deemed significantly enriched. To explore protein interactomes, a protein–protein interaction (PPI) network was constructed via the STRING database (v12.0) and clustered through the Markov Cluster Algorithm (inflation parameter = 1.5).

To elucidate multi‐omics interplay, a sequential integration strategy was employed. Data Integration Analysis for Biomarker discovery using Latent components (DIABLO) was applied to evaluate sample discrimination and extract top cross‐omics features. Integrated KEGG pathway analysis and Pearson correlation analysis (*p* < 0.05 was considered correlated) were employed to explore candidate molecules and potential mechanistic hypotheses.

### 2.6 Targeted Quantitative Validation and Correlation Analysis

To independently validate the high‐throughput multi‐omics findings and assess their clinical relevance, targeted quantitative assays were performed in the validation cohort (*n* = 8 per group). The absolute concentration of the candidate protein was quantified using a commercial Sandwich‐ELISA kit (Cusabio, China). Concurrently, absolute quantification of the correlated metabolites was strictly conducted using a targeted LC–MS/MS approach with specific authentic standards, achieving the highest identification confidence (MSI Level 1).

Furthermore, to evaluate the potential impact of clinical heterogeneity, Spearman correlation analyses were performed between the validated absolute molecular concentrations and continuous clinical covariates (including baseline CSF laboratory indices, post‐injury duration, and AIS grade), as well as between the candidate markers themselves.

Detailed experimental protocols, calibration parameters, and correlation statistical settings are provided in the Supporting Methods and Figure S1.

### 2.7 Statistical Analysis

Statistical analyses were performed using R (v4.3.1). Clinical data were compared using the Student's *t*‐test for normally distributed continuous variables, the Mann–Whitney *U* test for non‐normal distributions, and Fisher's exact test for categorical variables. For the untargeted multi‐omics profiling data, statistical significance was assessed using Student's *t*‐tests. For the targeted quantitative assays (ELISA and LC–MS/MS), unpaired *t*‐tests with Welch's correction were applied to strictly account for potential unequal variances between groups. A *p*‐value < 0.05 was considered statistically significant.

## 3 Results

### 3.1 Baseline Characteristics

The two groups within both the discovery (*n* = 10) and validation (*n* = 16) cohorts were well‐matched regarding age, sex, and comorbidities (*p* > 0.05; Table 1 and Table S1). Detailed injury characteristics for TSCI patients across both cohorts are provided in Table 2. Notably, baseline CSF analysis in the SCI group revealed significantly elevated red blood cell (RBC) counts and total protein concentrations (*p* < 0.05), indicating micro‐hemorrhage and blood‐spinal cord barrier (BSCB) disruption. Concurrently, plasma coagulation profiles indicated a hypercoagulable state following TSCI, as evidenced by shortened activated partial thromboplastin times (APTT) and increased D‐dimer (*p* < 0.05).

**TABLE 1:** Baseline characteristics of the discovery cohort.

Characteristic	Total (*n* = 10)	CON group (*n* = 5)	SCI group (*n* = 5)	*p*
*Demographics*				
Age	52.6 ± 15.47	54.40 ± 16.10	50.8 ± 16.28	0.64
Male sex	7 (70.0%)	3 (60.0%)	4 (80.0%)	1.00
*Comorbidities*				
Hypertension	3 (30%)	2 (40%)	1 (20%)	1.00
Diabetes	2 (20%)	1 (20%)	1 (20%)	1.00
Hyperlipidemia	2 (20%)	1 (20%)	1 (20%)	1.00
*Clinical measures*				
CSF RBC count (10^6^/L)	1645.50 [0.75, 5905.25]	0.00 [0.00, 3.00]	5963.00 [5732.00, 6933.00]	0.01*
CSF WBC count (10^6^/L)	11.00 [0.00, 40.00]	10.00 [0.00, 40.00]	12.00 [0.00, 25.00]	0.30
CSF glucose concentration (mmol/L)	3.40 ± 0.57	3.41 ± 0.68	3.41 ± 0.35	0.95
CSF protein concentration (mg/L)	2410.70 [705.73, 5749.98]	607.60 [270.60, 1000.10]	6138.2 [4585.30, 7723.80]	0.01*
Blood RBC count (10^12^/L)	3.69 ± 0.97	4.01 ± 0.55	3.37 ± 1.25	0.34
Blood WBC count (10^9^/L)	9.48 [6.02, 13.3]	6.17 [5.41, 9.56]	12.64 [10.14, 14.35]	0.10
Platelet count (10^9^/L)	182.00 ± 55.84	194.40 ± 66.29	169.60 ± 47.29	0.52
Serum glucose concentration (mmol/L)	5.92 [5.81, 7.66]	5.81 [4.33, 5.96]	7.66 [5.92, 13.11]	0.21
Serum ALT concentration (U/L)	25.00 [20.00, 42.00]	42.00 [25.00, 64.00]	20.00 [19.50, 20.00]	0.68
Serum AST concentration (U/L)	46.60 ± 37.13	27.60 ± 14.22	65.60 ± 44.69	0.13
Serum creatinine concentration (μmol/L)	66.10 ± 26.68	51.60 ± 25.56	80.60 ± 21.51	0.09
BUN concentration (mmol/L)	6.11 ± 2.78	5.34 ± 2.18	6.88 ± 3.31	0.41
PT (seconds)	11.66 ± 1.42	11.94 ± 1.07	11.38 ± 1.83	0.96
APTT (seconds)	27.75 [25.30, 31.70]	31.70 [30.60, 32.70]	25.30 [25.10, 25.40]	0.03*
Plasma D‐dimer (ug/L)	1844.50 [1581.00, 5539.00]	1597.00 [814.00, 1740.00]	5539.00 [1949.00, 6662.00]	0.01*

*Note:* An asterisk (*) denotes a *p*‐value < 0.05. Categorical variables are presented as number (percentage), normally distributed continuous variables as mean ± standard deviation, and non‐normally distributed variables as median [interquartile range].

Abbreviations: ALT, alanine aminotransferase; APTT, activated partial thromboplastin time; AST, aspartate aminotransferase; BUN, blood urea nitrogen; PT, prothrombin time; RBC, red blood cell; WBC, white blood cell.

**TABLE 2:** Injury conditions of patients with acute cervical traumatic spinal cord injury.

Patient ID	Age (years)	Etiology	Time post‐injury (hours)	Injury level	AIS grade
*Discovery Cohort*					
SCI1	50	Vehicle accident	38	C5‐C7	A
SCI2	62	Fall	15	C2‐C4	A
SCI3	50	Fall	24	C2‐C4	B
SCI4	71	Fall	17	C3‐C6	A
SCI5	22	Vehicle accident	20	C6‐C7	A
*Validation Cohort*					
SCI6	59	Vehicle accident	16	C5‐C6	A
SCI7	68	Fall	28	C4‐C7	A
SCI8	62	Vehicle accident	24	C4‐C6	B
SCI9	75	Fall	22	C2‐C3	A
SCI10	46	Vehicle accident	20	C2‐C6	A
SCI11	30	Vehicle accident	15	C3‐C5	A
SCI12	55	Fall	30	C3‐C4	A
SCI13	62	Vehicle accident	24	C4‐C6	B

Abbreviations: AIS, American spinal injury association impairment scale; SCI, spinal cord injury.

### 3.2 Proteomic Characterization of the Acute CSF Microenvironment

Our 4D‐DIA label‐free proteomic workflow quantified a total of 2905 proteins in the CSF (Figure S2). Principal component analysis (PCA) of the comprehensive dataset revealed a discernible separation between the SCI and CON groups (PC1 = 26.27%, PC2 = 20.35%; Figure 1A). Subsequent differential expression analysis identified 216 DEPs, comprising 102 upregulated and 114 downregulated proteins in the SCI group (Figure 1C; Figures S3 and S4). Unsupervised hierarchical clustering achieved apparent segregation of the TSCI and control samples (Figure 1B). Notably, Tenascin‐X (TNXB) and F‐box only protein 2 (FBXO2) were the top upregulated proteins by statistical significance and fold change, respectively, while splicing factor component U1 small nuclear ribonucleoprotein A (SNRPA) emerged as the most significantly downregulated protein (Figure 1C; Tables S2 and S3).

**FIGURE 1:**
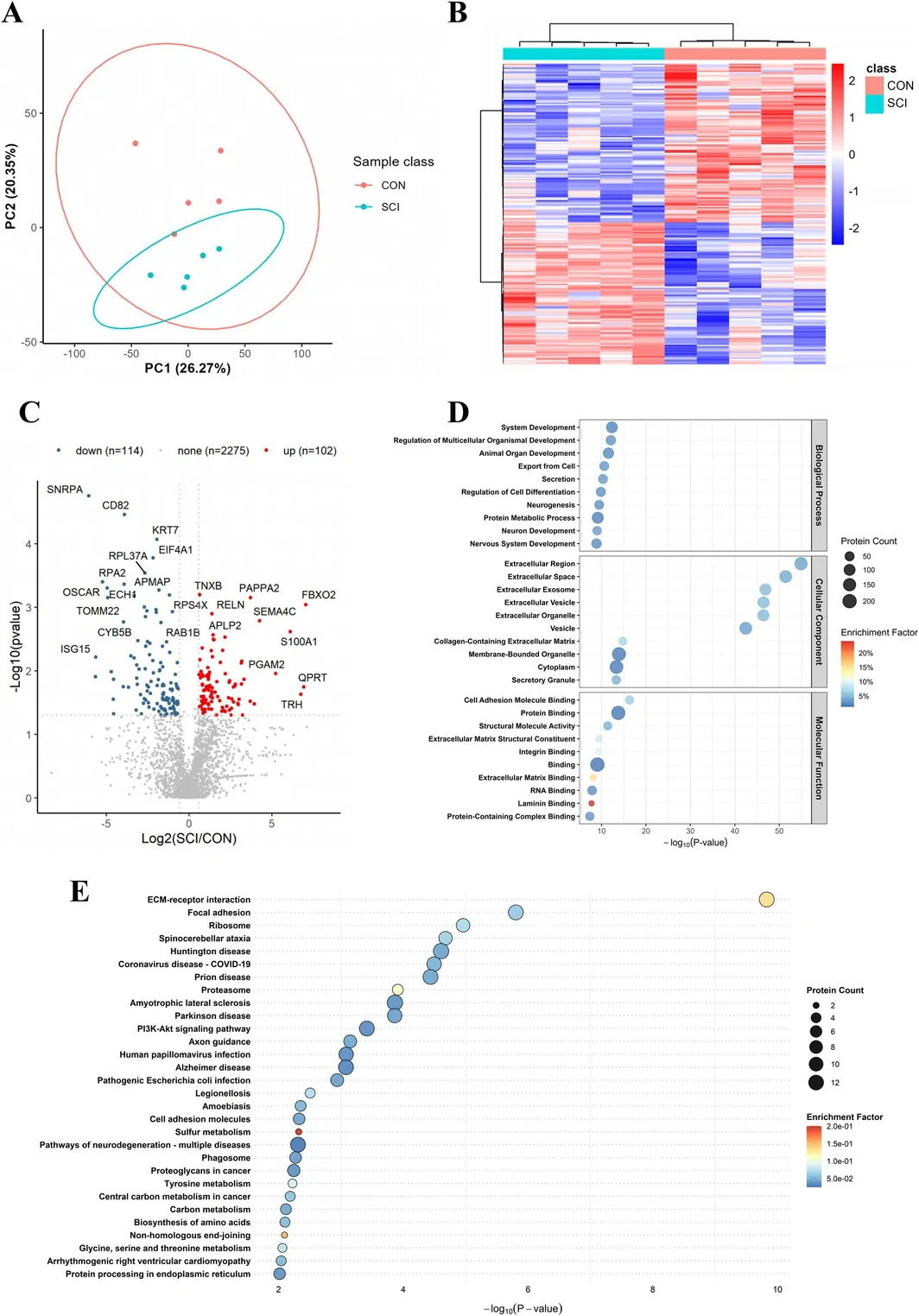
Proteomics analysis of CSF from the TSCI and control participants. (A) Principal Component Analysis (PCA) plot of the proteomic profiles, showing separation between the spinal cord injury (SCI, cyan) and control (CON, red) groups. (B) Unsupervised hierarchical clustering heatmap of differentially expressed proteins (DEPs) segregating the two groups. The fully labeled heatmap detailing protein identities is provided in Figure S3. (C) Volcano plot visualizing DEPs. 102 upregulated (red) and 114 downregulated (blue) proteins are highlighted (*p* < 0.05, FC > 1.5 or < 0.67). Individual‐level distribution plots of the top 10 upregulated and downregulated DEPs are provided in Figure S4. (D) Gene Ontology (GO) enrichment analysis of DEPs, displaying the top 10 significant terms for Biological Process (BP), Cellular Component (CC), and Molecular Function (MF). (E) KEGG pathway enrichment analysis, showing the top 30 most significantly enriched pathways (*p* < 0.05). For both (D) and (E), the *x*‐axis represents statistical significance (−log10(*p*‐value)), the bubble size corresponds to the count of mapped DEPs, and the color gradient indicates the enrichment factor.

To further elucidate the biological implications of these molecular alterations, GO analysis was performed, revealing intense tissue remodeling dominated by terms such as ‘extracellular matrix’, ‘extracellular vesicle’ and ‘cell adhesion’ (Figure 1D). These findings were reinforced by KEGG pathway analysis, which identified ‘ECM‐receptor interaction’ and ‘Focal adhesion’ as the top enriched pathways (Figure 1E). Furthermore, PPI network analysis pinpointed the ‘Collagen‐containing extracellular matrix’ cluster as the most densely interconnected functional module (Figure S5). Taken together, these convergent findings provide supporting evidence that extensive remodeling of the ECM is a dominant pathological feature in the CSF of acute TSCI.

### 3.3 Metabolomic Signatures of the Acute CSF Microenvironment

Global quality control evaluation demonstrated the high mass accuracy and MS/MS spectral matching reliability of our dataset (Figure S6A,B). Our untargeted metabolomics workflow initially detected 15,381 metabolic features, of which 602 were putatively identified via MS2 matching against the HMDB and KEGG databases (Figure S7). While the initial PLS‐DA exhibited clear group separation (*R* ^2^Y = 0.998, *Q* ^2^ = 0.832; Figure 2A), its permutation profile indicated a potential vulnerability to unfiltered orthogonal variance (Figure S8). To address this, an Orthogonal PLS‐DA (OPLS‐DA) model was applied to filter orthogonal noise. This upgraded model showed reliable predictive capacity (*R* ^2^Y = 0.998, *Q* ^2^ = 0.769; Figure 2B) and passed a 100‐permutation test (Figure 2C), indicating statistical validity without severe overfitting.

**FIGURE 2:**
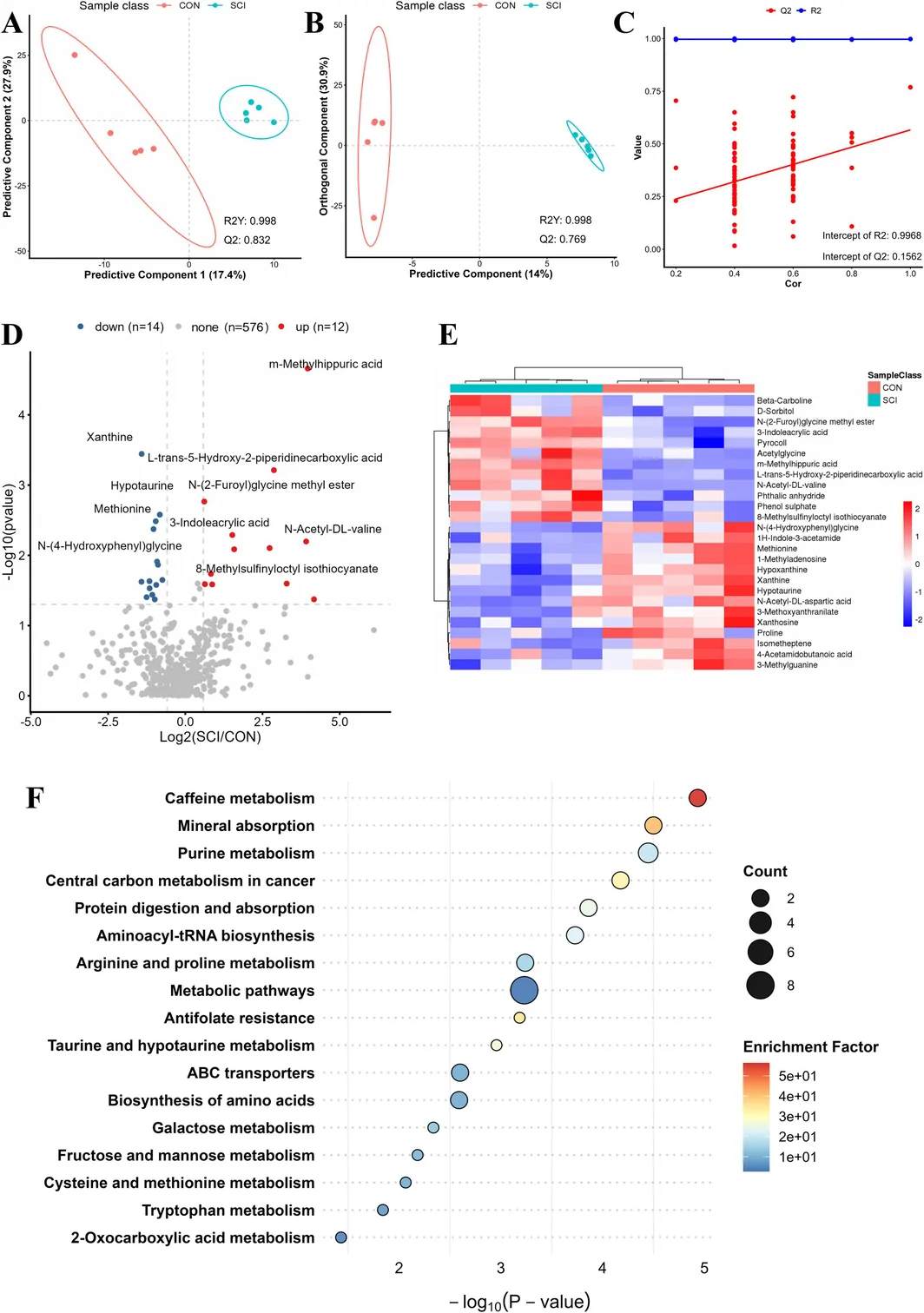
Metabolomics analysis of CSF from the TSCI and control participants. (A) Partial Least Squares Discriminant Analysis (PLS‐DA) score plot displaying global group distribution between the SCI (cyan) and CON (red) groups (*R* ^2^Y = 0.998, *Q* ^2^ = 0.832). (B) Orthogonal Partial Least Squares Discriminant Analysis (OPLS‐DA) score plot showing group discrimination after filtering orthogonal noise (*R* ^2^Y = 0.998, *Q* ^2^ = 0.769). (C) Permutation test plot (*n* = 100 iterations) for the OPLS‐DA model, showing R2 (blue) and Q2 (red) values from permuted class labels plotted against correlation coefficients between original and permuted vectors, with calculated *y*‐intercepts (R2 intercept = 0.997, Q2 intercept = 0.156). (D) Volcano plot of MS2‐confirmed metabolites. 12 upregulated (red) and 14 downregulated (blue) metabolites are highlighted (*p* < 0.05, FC > 1.5 or < 0.67, OPLS‐DA VIP > 1). Individual‐level distribution plots of the top 10 upregulated and downregulated DEMs are provided in Figure S9. (E) Unsupervised hierarchical clustering heatmap of differentially expressed metabolites (DEMs). (F) KEGG pathway enrichment analysis of DEMs, showing all the significantly enriched pathways (*p* < 0.05). The *x*‐axis represents statistical significance (−log10(*p*‐value)), the bubble size corresponds to the count of mapped DEMs, and the color gradient indicates the enrichment factor.

Based on our composite criteria, we identified 26 DEMs, comprising 12 upregulated and 14 downregulated metabolites in the TSCI cohort (Figure 2D and Figure S9). Unsupervised hierarchical clustering distinctly segregated the two groups (Figure 2E). Detailed inspection revealed a prominent downregulation of purine derivatives and key amino acids following TSCI. Specifically, xanthine represented the top downregulated metabolite, accompanied by essential amino acids such as methionine and proline, whereas m‐methylhippuric acid emerged as the top upregulated metabolite (Table S4 and Figure 2D).

To further delineate the implicated metabolic networks, KEGG pathway enrichment analysis was conducted. The top enriched pathways comprised ‘Caffeine metabolism’, ‘Mineral absorption’, and ‘Purine metabolism’, alongside key amino acid cascades such as ‘Arginine and proline metabolism’ and ‘Cysteine and methionine metabolism’ (Figure 2F). Collectively, these findings underscore widespread disruptions of purine and amino acid homeostasis within the CSF microenvironment post TSCI.

### 3.4 Multi‐Omics Integration and Identification of a GOT2‐Associated Metabolic Signature

To integrate the proteomic and metabolomic datasets and extract cross‐omics signatures, we applied the DIABLO framework, which differentiated TSCI from control samples (Figure 3A) and extracted the top features driving this separation (Figure 3B). Additionally, global Pearson correlation clustering revealed highly synchronized expression patterns across DEPs and DEMs, suggesting a broadly coordinated molecular response to injury (Figure 3C).

**FIGURE 3:**
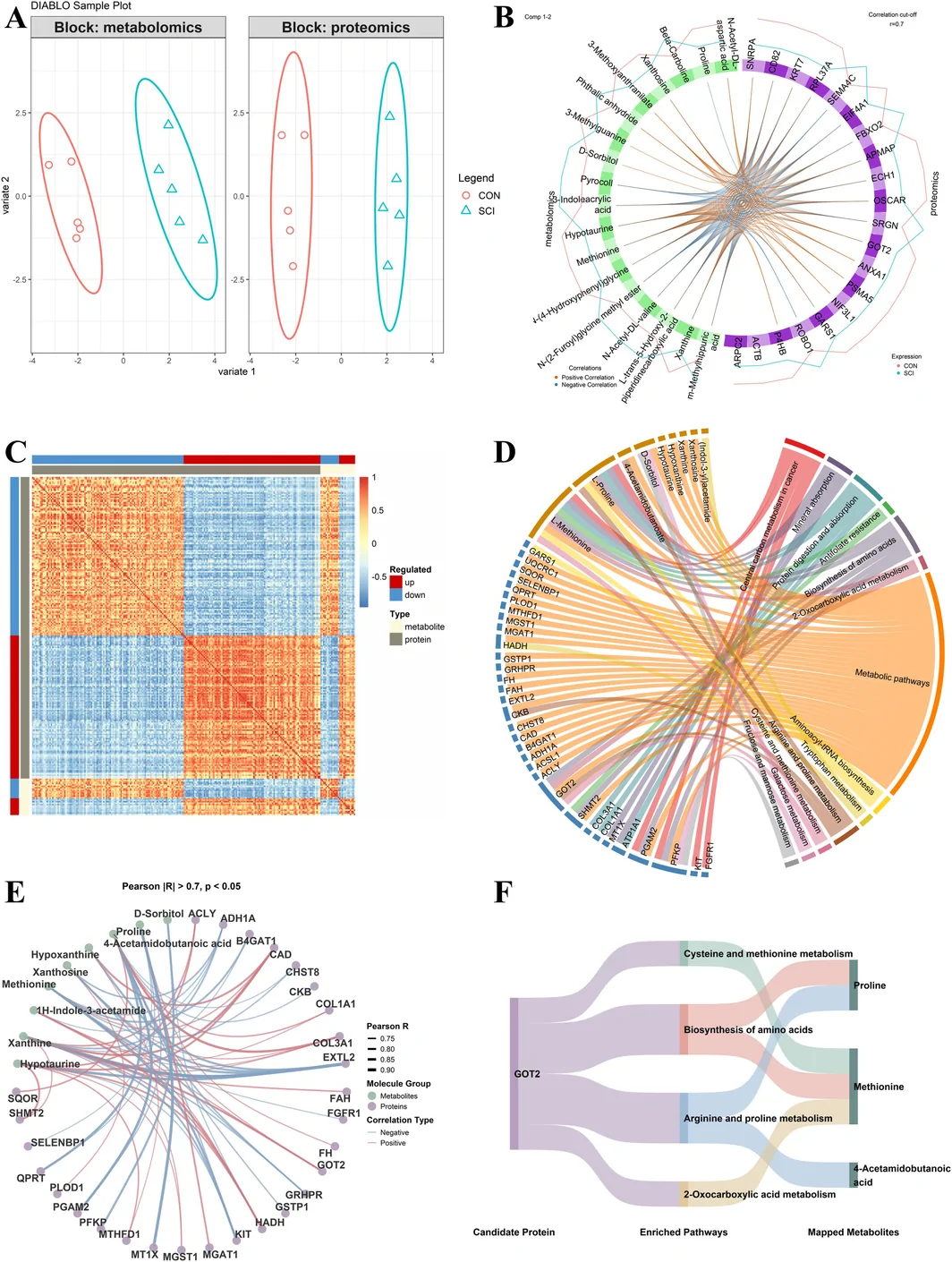
Integrated multi‐omics analysis identifies a GOT2‐Associated Metabolic Signature. (A) DIABLO sample plot displaying the discrimination between the SCI (cyan) and CON (red) groups based on the integrated proteomic and metabolomic profiles. (B) DIABLO circos plot highlighting the top highly correlated cross‐omics features contributing to the group separation. Proteins are indicated in purple and metabolites in green. (C) Pearson correlation heatmap showing global co‐expression patterns between all DEPs and DEMs (red: positive; blue: negative). (D) Chord diagram of the integrated KEGG pathway analysis, linking the shared biological processes (right) with the involved DEPs and DEMs (left). (E) Intra‐pathway correlation network illustrating extensive co‐expression linkages among the 33 DEPs and 9 DEMs identified within the shared KEGG pathways (red: Positive; blue: Negative). (F) Sankey diagram depicting the pathway co‐localization mapping of the candidate protein GOT2. The flow illustrates the linkages from GOT2, through four enriched amino acid related pathways, to the mapped downstream metabolites (methionine, 4‐acetamidobutanoic acid, and proline).

To uncover the biological functions underpinning these coordinated changes, integrated KEGG pathway analysis was performed. This analysis identified 13 significantly enriched pathways shared by both datasets (Figure 3D and Table S5), primarily centered on amino acid metabolism, including ‘Arginine and proline metabolism’, ‘Cysteine and methionine metabolism’, and ‘Tryptophan metabolism’. This shared enrichment highlighted amino acid metabolic reprogramming as a hallmark of acute TSCI pathophysiology.

Focusing on these shared pathways, a subset of 33 DEPs and 9 DEMs was identified, among which mitochondrial aspartate aminotransferase (GOT2) emerged as the highest‐frequency protein node (Figure 3D). To explore the interactions within this subset, an intra‐pathway correlation network (|*R*| > 0.7, *p* < 0.05, Figure 3E) was constructed, illustrating extensive co‐expression linkages. Within this network, the candidate protein GOT2 exhibited significant correlations with multiple nodes. To further visualize pathway co‐localization, a Sankey diagram mapped GOT2 to specific amino acid‐related pathways sharing methionine, 4‐acetamidobutanoic acid, and proline (Figure 3F).

To explicitly evaluate the relationships between GOT2 and these co‐localized metabolites, targeted Pearson correlation analysis was conducted. This revealed positive correlations between GOT2 and both methionine (*R* = 0.73, *p* = 0.018; Figure 4A) and 4‐acetamidobutanoic acid (*R* = 0.72, *p* = 0.019; Figure 4B). In contrast, proline lacked a statistically significant correlation (*p* > 0.05; Figure S10) despite its pathway co‐localization and was therefore excluded. Moreover, the relative abundances of these three interconnected candidate molecules (GOT2, methionine, and 4‐acetamidobutanoic acid) were consistently downregulated in the TSCI patients (Figure 4C–E). These multi‐omics observations were summarized in a hypothetical schematic (Figure 4F).

**FIGURE 4:**
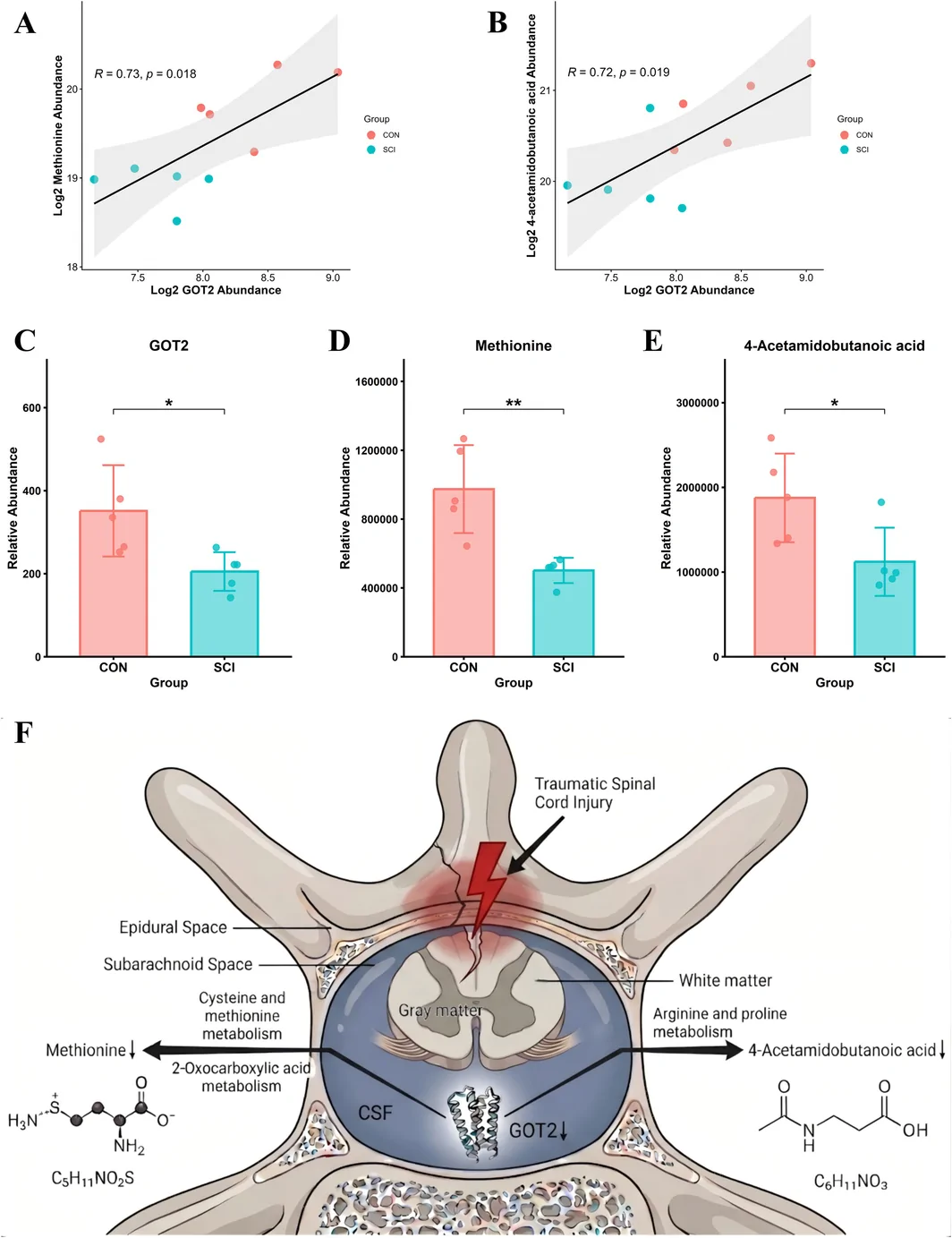
Selective correlation and concurrent downregulation of GOT2 and specific co‐mapped metabolites. (A, B) Pearson correlation scatter plots assessing the linear relationships between the relative abundance of GOT2 and (A) methionine, and (B) 4‐acetamidobutanoic acid. The R value represents the Pearson correlation coefficient, and the shaded areas indicate the 95% confidence intervals. (C–E) Bar‐scatter plots comparing the relative abundances of (C) GOT2, (D) methionine, and (E) 4‐acetamidobutanoic acid between the CON and SCI groups (*n* = 5 per group). Data are presented as mean ± SD. *p < 0.05, **p < 0.01. (F) A hypothetical schematic model illustrating the potential associations, wherein the downregulation of GOT2 following cervical traumatic spinal cord injury (TSCI) correlates with the coordinated decrease of specific co‐mapped metabolites across shared amino acid‐related pathways.

### 3.5 Targeted Validation of the GOT2‐Associated Metabolic Signature and Clinical Covariate Correlations

To independently validate the multi‐omics findings, we quantified the absolute concentrations of the candidate protein GOT2 and its co‐mapped metabolites (methionine and 4‐acetamidobutanoic acid) in CSF samples from the validation cohort (*n* = 16). Consistent with our high‐throughput profiling, quantitative analyses displayed a significant downregulation of GOT2 (*p* = 0.0024; Figure 5A; Table S6; Figure S11A,B) in the TSCI patients compared to controls. Concurrently, the concentrations of both methionine (*p* = 0.019; Figure 5B; Table S7; Figure S12A) and 4‐acetamidobutanoic acid (*p* = 0.014; Figure 5C; Table S7; Figure S12B) were significantly decreased in the CSF of TSCI patients.

**FIGURE 5:**
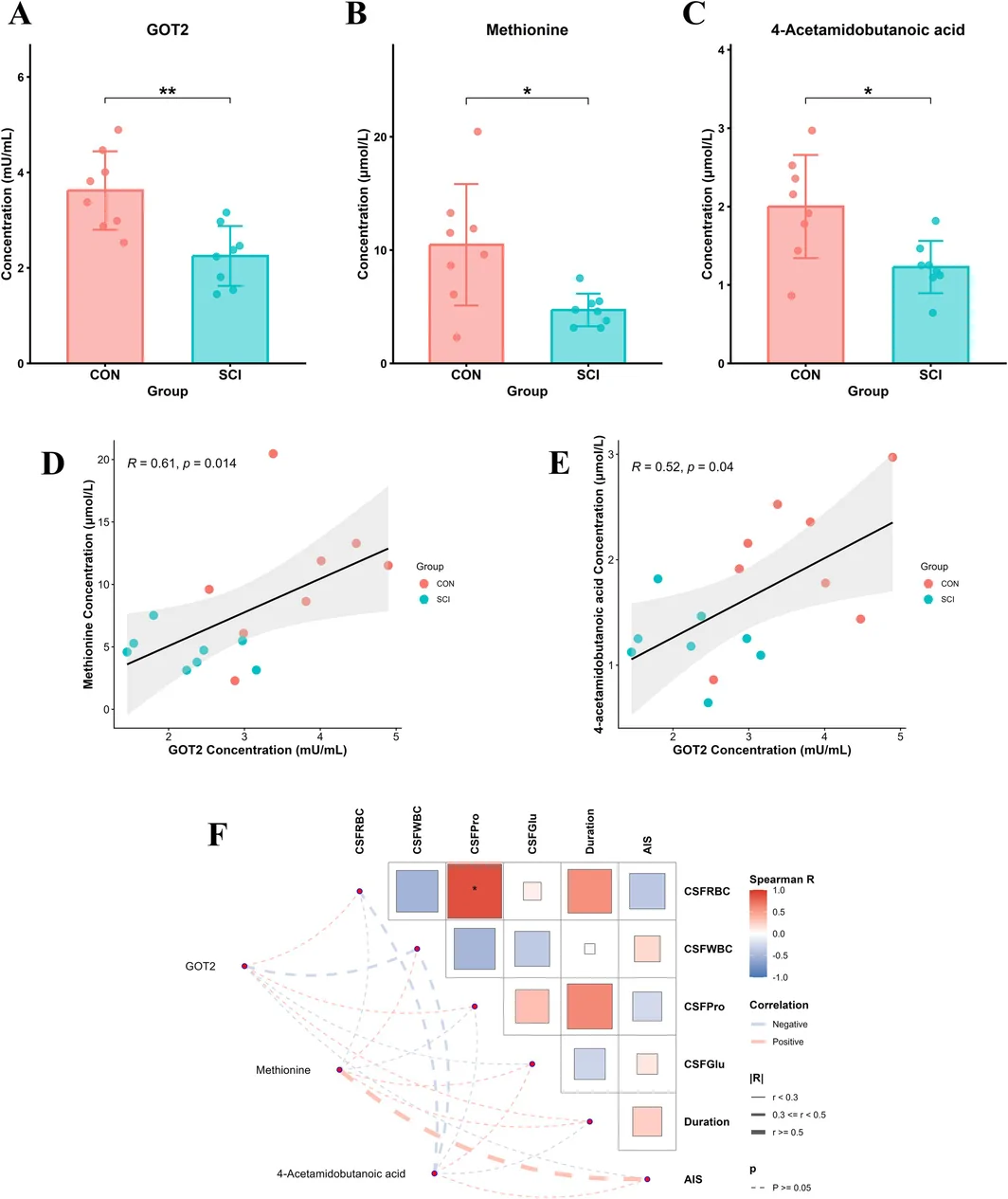
Targeted quantitative validation of the identified molecular signature and clinical covariate correlation analysis. (A–C) Absolute concentrations of (A) GOT2, (B) methionine, and (C) 4‐acetamidobutanoic acid in the CSF of the independent validation cohort (*n* = 16; 8 TSCI vs. 8 controls). Data are presented as mean ± SEM. Statistical differences were evaluated using an unpaired t‐test with Welch's correction (*p < 0.05, **p < 0.01). (D, E) Spearman correlation plots displaying the relationships between the concentration of GOT2 and (D) methionine, as well as (E) 4‐acetamidobutanoic acid. The solid lines represent linear regression fits, with shaded areas indicating the 95% confidence intervals. (F) Clinical covariate correlation network constructed to assess the potential influence of bleeding artifacts and clinical heterogeneity. The right‐hand matrix displays Spearman correlation coefficients among clinical parameters using a color gradient (red for positive correlations, blue for negative correlations). The left‐hand network maps the correlations between the validated molecular markers and these clinical indices, with dashed edges denoting statistically non‐significant associations (*p* ≥ 0.05).

To further investigate the associations proposed by our multi‐omics integration network, we assessed the relationships between the concentrations of these markers. Spearman correlation analyses showed a significant positive correlation between the concentrations of GOT2 and methionine (*R* = 0.61, *p* = 0.014; Figure 5D), as well as between GOT2 and 4‐acetamidobutanoic acid (*R* = 0.52, *p* = 0.04; Figure 5E).

Finally, a clinical covariate correlation network was constructed within the TSCI cohort to assess the potential influence of bleeding artifacts and clinical heterogeneity (Figure 5F). As anticipated in acute neurotrauma, a significant positive correlation was exclusively observed between CSF RBC count and CSF total protein. Notably, the absolute concentrations of GOT2, methionine, and 4‐acetamidobutanoic acid exhibited no significant correlations with the CSF laboratory indices, post‐injury duration, or AIS grade (all *p* > 0.05).

## 4 Discussion

High‐throughput omics technologies have significantly advanced our understanding of the molecular cascade following TSCI, yet the field has relied heavily on preclinical animal models, primarily in rodents [13, 17]. While invaluable for mechanistic dissection, these models may not fully recapitulate the complex genetic and physiological background of human TSCI [17, 18]. Compared to peripheral blood, CSF is insulated by the blood‐spinal cord barrier, offering a more faithful reflection of the central pathological state [19, 20]. Furthermore, unlike tissue biopsies which capture a static and spatially limited view, CSF provides a dynamic readout of biochemical events [21].

Despite these advantages, human CSF‐based omics studies in acute TSCI remain rare due to logistical challenges regarding sample collection [22], with no integrated multi‐omics investigations reported to date. To address this void, we presented an integrated proteomic and metabolomic analysis of human CSF during the acute phase of cervical TSCI. Our preliminary exploratory study revealed profound molecular reprogramming of the CSF microenvironment, providing a system‐wide view of human TSCI.

Evidence from animal models has shown that following TSCI, the composition and structure of ECM undergo extensive remodeling, implicating both tissue repair and inhibitory scar formation [23, 24]. Our data provide observational insights into this structural response in the human setting. The primary mechanical injury inflicts immediate physical damage to the spinal cord tissue, causing direct disruption of the ECM architecture [25]. This event releases numerous ECM fragments, such as hyaluronan, Tenascin proteins, and sulfated proteoglycans [23]. The prominent upregulation of Tenascin‐X (TNXB) in our study indicates a substantial release of ECM components, which may trigger innate immune infiltration, potentially perpetuating a vicious cycle of inflammation and ECM degradation [23].

Moreover, the ECM plays an essential role in scar formation throughout the subacute and chronic stages [26]. As the acute inflammatory response subsides, the body initiates a wound healing process, ultimately forming dense scar tissue [27]. The scar formed after TSCI has a significant dual role. On one hand, it acts as a neuroprotective barrier, confining inflammation, excitotoxicity, and oxidative stress to the lesion core. On the other hand, it forms a physical and chemical barrier to axon regeneration [27]. This contradiction underscores the need for precise timing in therapeutic interventions, which may explain why few ECM‐targeting therapies have attained clinical translation [24, 28]. Our human CSF findings further emphasize the prominence of ECM remodeling during the acute phase of human TSCI, suggesting that early‐phase stabilization of the ECM structure might preempt downstream destructive cascades.

Beyond structural changes, our data reveal a state of metabolic reprogramming. The observed dysregulation of purine and amino acid metabolism aligns with recent reports from rodent models [29, 30], underscoring their potential relevance in the acute metabolic response. Consistent with our results, Zeng et al. reported synchronous disruptions in the ‘Purine metabolism’ pathway at both transcriptomic and metabolomic levels in a rat TSCI model [29]. Since purine metabolism is linked to the synthesis of ATP and reactive oxygen species (ROS) [31], the alteration of this pathway, evidenced by the downregulation of xanthine and hypoxanthine, points towards potential disorders in cellular energy homeostasis and oxidative stress management. Given that oxidative stress is a hallmark of secondary injury, these alterations may aggravate the extent of tissue damage [13]. While our analysis also identified ‘Caffeine metabolism’ as the most enriched pathway, we posit this likely reflects the broader purine pathway disruption rather than a specific response to caffeine itself, particularly as caffeine was not detected in the CSF.

Concurrently, pathway enrichment analysis of DEMs highlighted the dysregulation of multiple amino acid metabolism‐related pathways as a prominent feature of acute TSCI. In addition, by co‐mapping the proteomic and metabolomic datasets onto KEGG pathways, our integrated analysis provided a unified view of these molecular alterations, identifying several amino acid metabolic pathways as significantly perturbed biological processes. These findings extend beyond single‐molecule identification to imply a systemic disturbance in fundamental metabolic processes. Similar disturbances have been documented following CNS trauma, often characterized by the depletion of critical amino acids [32, 33, 34]. Based on these reports, such depletion may be involved in a pathogenic cascade culminating in reduced antioxidant capacity and sustained neuroinflammation, thereby potentially participating in the secondary injury process.

A prominent functional consequence of this pathway‐level disruption is the dysregulation of essential amino acids and their derivatives. Notably, we observed a significant downregulation of methionine, proline, and the Gamma‐aminobutyric acid (GABA) derivative 4‐acetamidobutanoic acid. The downregulation of methionine, an initiator of protein synthesis and precursor for the antioxidant glutathione (GSH), suggests a possible vulnerability in tissue repair and oxidative stress defense [35]. Similarly, the decrease of proline, an essential component of collagen, may reflect alterations in structural repair mechanisms [36], while the reduction in 4‐acetamidobutanoic acid implies potential dysregulation of inhibitory neurotransmission [37]. Collectively, such widespread amino acid downregulation may represent not merely a passive manifestation of injury but potentially a contributing factor to the secondary injury cascade, though this remains to be experimentally verified.

Initial DIABLO and correlation clustering analyses displayed relevant TSCI discrimination and extensive cross‐omics interplay. Within this coordinated response, our integrated KEGG analysis highlighted GOT2 as a candidate marker exhibiting the highest connectivity to dysregulated metabolic pathways, particularly those involving amino acid metabolism. This computational finding was supported by ELISA analysis, which demonstrated a significant downregulation of GOT2 in the CSF of TSCI patients. This observation aligns with previous findings in traumatic brain injury (TBI), where reduced GOT2 was reported in mitochondrial fractions of the cerebral cortex [38]. This concordance across different CNS trauma models supports the notion that GOT2 may play an important role in neurotrauma.

Biologically, GOT2 is a crucial enzyme of cellular energy metabolism, serving as a core component of the malate–aspartate shuttle (MAS) and glutamine metabolism [39]. By catalyzing the reversible conversion of glutamate and oxaloacetate, it regulates the mitochondrial NAD^+^/NADH balance essential for ATP production [39]. This critical metabolic role is underscored by experimental evidence in TBI [38], where Arun et al. demonstrated that the downregulation of GOT2 contributes to impaired mitochondrial function and ATP deficits following neurotrauma. Consequently, we speculate that the observed downregulation of GOT2 in the CSF might reflect a state of altered mitochondrial metabolism at the injury site, potentially paralleling the bioenergetic failure characteristic of acute TSCI.

To explore the functional implications of GOT2 downregulation, we analyzed its correlation with linked metabolites. Our multi‐omics analyses demonstrated that GOT2 positively correlates with methionine and 4‐acetamidobutanoic acid, but not with proline despite their pathway co‐localization. This suggests that the metabolic alterations associated with GOT2 may be selective, potentially involving specific targets rather than globally affecting all pathway‐connected substrates. Notably, both GOT2 and methionine were previously highlighted among the top discriminative features in our DIABLO model, providing cross‐methodological support for this potential metabolic signature.

Crucially, these multi‐omics findings were successfully reproduced in the independent validation cohort. Targeted quantitative assays displayed the significant downregulation of both methionine and 4‐acetamidobutanoic acid and showed their positive correlations with GOT2 concentrations, thereby supporting the reliability of the multi‐omics profiling. Since GOT2 regulates glutamate conversion [39], while methionine serves as a critical precursor for antioxidant GSH synthesis [35], their concurrent decrease might point towards a relationship with a compromised CNS antioxidant capacity in response to the post‐traumatic ROS surge [29]. Although the role of 4‐acetamidobutanoic acid in TSCI remains unreported, its potential functional relationship with GOT2 warrants future investigation.

Based on these multi‐omics data and existing literature, we hypothesize that GOT2 may play an important role in the secondary injury post TSCI. The significant downregulation of GOT2 might be associated with the decrease of specific protective metabolites, potentially bridging amino acid metabolic dysregulation to secondary injury processes. Direct evaluation of GOT2 enzymatic activity, mitochondrial respiration, and oxidative stress markers in preclinical models will be essential to validate these mechanistic hypotheses and explore feasible therapeutic strategies.

In evaluating this acute CSF microenvironment, an important technical consideration is the potential influence of intramedullary hemorrhage and blood‐spinal cord barrier disruption. It is worth noting that while raw clinical CSF exhibited elevated RBCs and total protein, strict pre‐analytical centrifugation effectively minimized technical blood contamination. A comprehensive sensitivity evaluation of our multi‐omics networks demonstrated the absence of classical plasma or erythrocyte contamination markers (e.g., albumin, hemoglobin, or heme) among the DEPs and DEMs. Coupled with our targeted clinical correlation findings, this supports the notion that the identified multi‐omics signature primarily reflects an intrinsic cellular metabolic alteration rather than a blood‐derived artifact.

Another crucial aspect to evaluate is the potential impact of clinical heterogeneity. By strictly limiting our enrollment to severe cervical TSCI cases (AIS A and B) within 48 h post‐injury, we aimed to inherently minimize such variance. Furthermore, our targeted correlation analyses revealed that the concentrations of the validated molecules exhibited no significant correlation with CSF laboratory indices, post‐injury duration, or baseline AIS grade. While this suggests the extracted signature may reflect a consistent metabolic response within the hyper‐acute window, the absence of detectable correlation in a limited cohort does not definitively exclude potentially subtle but meaningful associations.

While this study provides novel insights into the molecular landscape of human TSCI, several limitations warrant consideration. First, our sample size was limited by the logistical challenges of CSF collection from TSCI individuals, which necessitated the use of nominal *p*‐values during initial omics screening to preserve discovery breadth. Lacking prior multi‐omics datasets for human TSCI CSF, an a priori sample size calculation was unfeasible. Thus, enrollment balanced sample availability with strict inclusion criteria to minimize clinical heterogeneity. Although these criteria and subsequent experimental validations mitigate the risk of false positives, the identified signatures should be considered exploratory and require future validation in larger cohorts. Furthermore, as our current evaluations provide only a cross‐sectional snapshot within the hyper‐acute window, both the temporal evolution of these molecular responses across subsequent injury phases and their predictive value for long‐term neurological recovery warrant future longitudinal investigation.

Second, the utilization of iNPH patients as controls represents a necessary ethical compromise rather than a perfectly healthy baseline. To minimize potential confounding, we enforced strict inclusion criteria, such as requiring normal CSF biochemistries for controls and an intact dura mater with rapid intraoperative sampling for TSCI patients. Furthermore, a literature‐based sensitivity analysis demonstrated negligible overlap with established iNPH proteomics signatures [40]. While this suggests injury‐induced proteomic signals predominate, potential metabolomic confounding from the iNPH baseline cannot be fully excluded.

Finally, our bulk multi‐omics approach captures molecular alterations but cannot resolve their cellular origin or establish causality. Therefore, future investigations employing single‐cell spatial transcriptomics are required to pinpoint the cellular sources of these metabolic shifts. Meanwhile, integrating these high‐resolution techniques with targeted mechanistic models will be essential to further elucidate the causal role of GOT2‐associated metabolic signature, which may provide a preliminary molecular basis for future TSCI research.

## 5 Conclusions

This exploratory multi‐omics analysis reveals a distinct molecular profile of the acute human TSCI microenvironment, characterized by extensive ECM remodeling and metabolic dysregulation. We identified GOT2 as a candidate marker, whose downregulation was correlated with the decrease of specific neuroprotective metabolites. These findings suggest that the GOT2‐associated metabolic signature warrants further mechanistic investigation regarding its pathogenic implications and potential therapeutic relevance.

## Author Contributions

W.J. and Y.W. have contributed equally to this work; H.C. and N.L. conceived this project, acquired funding, provided full guidance, and revised the manuscript; W.J. and Y.W. conducted sample curation, bioinformatics and statistical analyses, experimental validation, and wrote the manuscript; W.M., H.L., Q.Q., T.J., C.S., and Z.Z. assisted with sample curation and methodology in this study.

## Funding

This study was supported by Natural Science Foundation of Jiangsu Province (Grant No. BK20242099) and National Natural Science Foundation of China (Grant No. 82071393). The authors declare financial support was received for the research, authorship, and publication of this article.

## Ethics Statement

The study was performed in accordance with the ethical standards of the Declaration of Helsinki (1964) and its subsequent amendments [41]. This study was approved by the Ethics Committee of Zhongda Hospital, Southeast University (approval ID: 2024ZDSYLL469‐P01). Written informed consent was obtained from all participants or their legal guardians.

## Conflicts of Interest

The authors declare no conflicts of interest.

## Supporting information


**Figure S1:** Comprehensive multi‐omics study design and analytical workflow. The flowchart illustrates the sequential phases of the study, encompassing study cohort enrollment and CSF sampling (Step 1), high‐throughput multi‐omics data acquisition and processing (Step 2), downstream bioinformatics analysis and multi‐omics integration (Step 3), independent targeted validation of the identified signature (Step 4), and clinical contextualization (Step 5). Specific analytical parameters, data processing thresholds, quality control criteria, and software versions for each step are detailed within the respective panels. TSCI, traumatic spinal cord injury; iNPH, idiopathic normal pressure hydrocephalus; CSF, cerebrospinal fluid; DIA‐PASEF, data‐independent acquisition parallel accumulation serial fragmentation; DDA, data‐dependent acquisition; QC, quality control; CV, coefficient of variation; PQN, probabilistic quotient normalization; DEPs, differentially expressed proteins; DEMs, differentially expressed metabolites; PLS‐DA, partial least squares discriminant analysis; VIP, variable importance in projection; LOOCV, leave‐one‐out cross‐validation; BSCB, blood‐spinal cord barrier.
**Figure S2:** Overview of protein identification across all samples. The bar chart displays the number of proteins identified. The orange bar indicates the total number of unique proteins identified across the entire cohort (*n* = 10). Green and blue bars show the number of proteins identified in each individual CON and SCI sample, respectively.
**Figure S3:** Heatmap of all differentially expressed proteins (DEPs). Each row corresponds to one DEP, and each column represents an individual sample. The heatmap visualizes the *Z*‐score normalized abundance of each protein, with red indicating higher abundance and blue indicating lower abundance relative to the mean.
**Figure S4:** Box plots displaying the log2‐transformed abundances of the top 10 significantly upregulated (top two rows) and top 10 downregulated (bottom two rows) proteins in the SCI group (cyan, *n* = 5) compared to the CON group (red, *n* = 5). The horizontal line within each box represents the median, the lower and upper boundaries of the box indicate the first and third quartiles, respectively, and the whiskers extend to the minimum and maximum values. Individual data points are overlaid to display the complete sample distribution.
**Figure S5:** PPI network of DEPs. The network was constructed using the STRING database to visualize known and predicted interactions among the DEPs. Nodes represent proteins, and edges represent the associations between them. Nodes are colored based on functional clusters identified by the MCL algorithm. A legend at the bottom details the top 12 largest clusters, with the ‘Collagen‐containing extracellular matrix’ cluster (red) being the most prominent.
**Figure S6:** Global quality control metrics for untargeted metabolite identification. (A) Distribution of the mass error (Delta mass, in Daltons) for putatively identified features in negative (top) and positive (bottom) ionization modes. (B) Distributions of MS/MS spectral matching scores (Forward, Reverse, and Total scores) against standard reference libraries (HMDB and KEGG) for both ionization modes.
**Figure S7:** Overview of the metabolite identification workflow. The bar chart quantifies the number of metabolic signals detected at different stages of the identification process, shown for positive ion mode (orange), negative ion mode (blue), and their total (green).
**Figure S8:** Permutation test plot for the Partial Least Squares Discriminant Analysis (PLS‐DA) model (*n* = 100 iterations). The plot displays the distribution of permuted R2 (blue) and Q2 (red) values as a function of the correlation coefficient between the permuted and original class label vectors. The calculated y‐intercepts (R2 intercept = 0.996, Q2 intercept = 0.663) reflect a relatively high baseline Q2 value under standard PLS‐DA modeling. This indicates the model's inherent vulnerability to un‐filtered orthogonal variance in a small‐sample cohort, thereby proposing the requirement of applying subsequent orthogonal signal correction (OPLS‐DA) for reliable feature evaluation.
**Figure S9:** Box plots displaying the log2‐transformed intensities of the top 10 significantly upregulated (top two rows) and top 10 downregulated (bottom two rows) metabolites in the SCI group (cyan, *n* = 5) compared to the CON group (red, *n* = 5). The horizontal line within each box represents the median, the lower and upper boundaries of the box indicate the first and third quartiles, respectively, and the whiskers extend to the minimum and maximum values. Individual data points are overlaid to display the complete sample distribution.
**Figure S10:** Pearson correlation analysis between GOT2 and proline. Scatter plot illustrating the relationship between the Log2‐transformed proteomic abundance of GOT2 and the metabolomic abundance of proline in the discovery cohort (*n* = 10). In contrast to the positive linear correlation observed with methionine and 4‐acetamidobutanoate, no statistically significant correlation was identified for proline (*p* = 0.33). The R value represents the Pearson correlation coefficient, and the shaded area indicates the 95% confidence interval.
**Figure S11:** Standard curve and raw optical density map for the GOT2 ELISA. The standard curve was fitted using a rational function model: Y = (a + bX)/(1 + cX + dX^2^). The calculated parameters were: a = −0.02, b = 10.25, c = −1.51, d = −0.12. Goodness of fit: *r* > 0.99. Note that the unit mU/mL on the y‐axis represents kit‐specific concentration units quantifying structural protein mass, rather than functional enzyme activity. (B) Pseudocolor map of the raw optical density (OD) measured at 450 nm across the assay plate. The layout highlights the designated wells for the recombinant standards, blank controls, and clinical CSF samples from the CON and SCI groups.
**Figure S12:** Calibration curves for targeted absolute quantification of candidate metabolites. (A) Standard curve for L‐methionine. The linear regression equation is Y = 403.059X—5347.4 (*r* > 0.99). (B) Standard curve for 4‐acetamidobutanoic acid. The linear regression equation is Y = 2.98594X—22.8215 (*r* > 0.99). Both calibration curves were constructed using authentic external standards and fitted with a linear regression model applying a 1/X weighting factor. The x‐axis represents the nominal concentration of the standards (nmol/L), and the y‐axis represents the integrated MS peak area response.

**Data S1:** cns71242‐sup‐0002‐SupplementaryMethods.docx.

**Table S1:** Baseline characteristics of the independent validation cohort.

**Table S2:** The upregulated differentially expressed proteins sorted by *p*‐value (ascending).

**Table S3:** The downregulated differentially expressed proteins sorted by *p*‐value (ascending).

**Table S4:** Differentially expressed metabolites sorted by *p*‐value (ascending).

**Table S5:** Integrated proteometabolomic KEGG pathway analysis in cervical TSCI CSF.

**Table S6:** Raw data of ELISA validation of CSF GOT2 concentration.

**Table S7:** Raw data of targeted metabolomics validation.

**Table S8:** Processed quantitative matrices for proteomics data (relative abundance).

**Table S9:** Processed quantitative matrices for metabolomics data (relative abundance).

## Data Availability

The mass spectrometry proteomics data have been deposited to the ProteomeXchange Consortium (https://proteomecentral.proteomexchange.org) via the iProX partner repository with the dataset identifier PXD069500. The mass spectrometry metabolomics data have been deposited to the China National GeneBank Sequence Archive (https://db.cngb.org/cnsa/) with data set identifier CNP0008195. The custom R scripts for downstream statistical analysis and multi‐omics integration are available at GitHub (https://github.com/jwk770/TSCI‐CSF‐Multiomics.git). The processed quantitative matrices for both omics layers are provided as Tables S8 and S9, respectively.
