# Supplementary material for: Integrated Multi‐Omics Analysis of Human Cerebrospinal Fluid in Traumatic Spinal Cord Injury: An Exploratory Pilot Study
Source: CNS Neurosci Ther. 2026 Nov 2;32(11):e71242. doi: 10.1002/cns.71242 (PMC13726482; doi:10.1002/cns.71242)
Supplement: Supplementary file 1 [file CNS-32-e71242-s006.docx]

**Supplementary Figure 1.** Comprehensive multi-omics study design and analytical workflow. The flowchart illustrates the sequential phases of the study, encompassing study cohort enrollment and CSF sampling (Step 1), high-throughput multi-omics data acquisition and processing (Step 2), downstream bioinformatics analysis and multi-omics integration (Step 3), independent targeted validation of the identified signature (Step 4), and clinical contextualization (Step 5). Specific analytical parameters, data processing thresholds, quality control criteria, and software versions for each step are detailed within the respective panels. TSCI, traumatic spinal cord injury; iNPH, idiopathic normal pressure hydrocephalus; CSF, cerebrospinal fluid; DIA-PASEF, data-independent acquisition parallel accumulation serial fragmentation; DDA, data-dependent acquisition; QC, quality control; CV, coefficient of variation; PQN, probabilistic quotient normalization; DEPs, differentially expressed proteins; DEMs, differentially expressed metabolites; PLS-DA, partial least squares discriminant analysis; VIP, variable importance in projection; LOOCV, leave-one-out cross-validation; BSCB, blood-spinal cord barrier.

**Supplementary Figure 2.** Overview of protein identification across all samples. The bar chart displays the number of proteins identified. The orange bar indicates the total number of unique proteins identified across the entire cohort (n=10). Green and blue bars show the number of proteins identified in each individual CON and SCI sample, respectively.

**Supplementary Figure 3.** Heatmap of all differentially expressed proteins (DEPs). Each row corresponds to one DEP, and each column represents an individual sample. The heatmap visualizes the Z-score normalized abundance of each protein, with red indicating higher abundance and blue indicating lower abundance relative to the mean.

**Supplementary Figure 4.** Box plots displaying the log2-transformed abundances of the top 10 significantly upregulated (top two rows) and top 10 downregulated (bottom two rows) proteins in the SCI group (cyan, n=5) compared to the CON group (red, n=5). The horizontal line within each box represents the median, the lower and upper boundaries of the box indicate the first and third quartiles, respectively, and the whiskers extend to the minimum and maximum values. Individual data points are overlaid to display the complete sample distribution.

**Supplementary Figure 5.** PPI network of DEPs. The network was constructed using the STRING database to visualize known and predicted interactions among the DEPs. Nodes represent proteins, and edges represent the associations between them. Nodes are colored based on functional clusters identified by the MCL algorithm. A legend at the bottom details the top 12 largest clusters, with the ‘Collagen-containing extracellular matrix’ cluster (red) being the most prominent.

**Supplementary Figure 6.** Global quality control metrics for untargeted metabolite identification. (A) Distribution of the mass error (Delta mass, in Daltons) for putatively identified features in negative (top) and positive (bottom) ionization modes. (B) Distributions of MS/MS spectral matching scores (Forward, Reverse, and Total scores) against standard reference libraries (HMDB and KEGG) for both ionization modes.

**Supplementary Figure 7.** Overview of the metabolite identification workflow. The bar chart quantifies the number of metabolic signals detected at different stages of the identification process, shown for positive ion mode (orange), negative ion mode (blue), and their total (green).

**Supplementary Figure 8.** Permutation test plot for the Partial Least Squares Discriminant Analysis (PLS-DA) model (*n*=100 iterations). The plot displays the distribution of permuted R2 (blue) and Q2 (red) values as a function of the correlation coefficient between the permuted and original class label vectors. The calculated y-intercepts (R2 intercept = 0.996, Q2 intercept = 0.663) reflect a relatively high baseline Q2 value under standard PLS-DA modeling. This indicates the model's inherent vulnerability to un-filtered orthogonal variance in a small-sample cohort, thereby proposing the requirement of applying subsequent orthogonal signal correction (OPLS-DA) for reliable feature evaluation.

**Supplementary Figure 9.** Box plots displaying the log2-transformed intensities of the top 10 significantly upregulated (top two rows) and top 10 downregulated (bottom two rows) metabolites in the SCI group (cyan, n=5) compared to the CON group (red, n=5). The horizontal line within each box represents the median, the lower and upper boundaries of the box indicate the first and third quartiles, respectively, and the whiskers extend to the minimum and maximum values. Individual data points are overlaid to display the complete sample distribution.

**Supplementary Figure 10.** Pearson correlation analysis between GOT2 and proline. Scatter plot illustrating the relationship between the Log2-transformed proteomic abundance of GOT2 and the metabolomic abundance of proline in the discovery cohort (n=10). In contrast to the positive linear correlation observed with methionine and 4-acetamidobutanoate, no statistically significant correlation was identified for proline (*p*=0.33). The R value represents the Pearson correlation coefficient, and the shaded area indicates the 95% confidence interval.

**Supplementary Figure 11.** Standard curve and raw optical density map for the GOT2 ELISA. The standard curve was fitted using a rational function model: Y = (a + bX) / (1 + cX + dX^2^). The calculated parameters were: a = -0.02, b = 10.25, c = -1.51, d = -0.12. Goodness of fit: r > 0.99. Note that the unit mU/mL on the y-axis represents kit-specific concentration units quantifying structural protein mass, rather than functional enzyme activity. (B) Pseudocolor map of the raw optical density (OD) measured at 450 nm across the assay plate. The layout highlights the designated wells for the recombinant standards, blank controls, and clinical CSF samples from the CON and SCI groups.

**Supplementary Figure 12.** Calibration curves for targeted absolute quantification of candidate metabolites. (A) Standard curve for L-methionine. The linear regression equation is Y = 403.059X - 5347.4 (r>0.99). (B) Standard curve for 4-acetamidobutanoic acid. The linear regression equation is Y = 2.98594X - 22.8215 (r>0.99). Both calibration curves were constructed using authentic external standards and fitted with a linear regression model applying a 1/X weighting factor. The x-axis represents the nominal concentration of the standards (nmol/L), and the y-axis represents the integrated MS peak area response.
