# Supplementary material for: Integrated Multi‐Omics Analysis of Human Cerebrospinal Fluid in Traumatic Spinal Cord Injury: An Exploratory Pilot Study
Source: CNS Neurosci Ther. 2026 Nov 2;32(11):e71242. doi: 10.1002/cns.71242 (PMC13726482; doi:10.1002/cns.71242)
Supplement: Supplementary file 2 [file CNS-32-e71242-s010.docx]

Integrated Multi-omics Analysis of Human Cerebrospinal Fluid in Traumatic Spinal Cord Injury: an Exploratory Pilot Study

**Supplementary Methods**

**1.1 Omics Raw Data Processing and Normalization**

To minimize technical confounders and ensure high-quality data for downstream statistical evaluations, raw feature matrices generated from both proteomics and metabolomics pipelines were subjected to rigorous domain-specific data cleaning, imputation, and normalization protocols. All biological and Quality Control (QC) samples were extracted and acquired within a single analytical batch, and the instrumental injection sequence was completely randomized.

**1.1.1 Proteomics Data Processing**

Following raw data extraction and peptide-to-protein rollout via Spectronaut, a strict missingness threshold was enforced. Proteins present in less than 20% of the samples (i.e., missingness > 80% across the cohort) were excluded from the dataset. For the remaining matrix, missing values were imputed using 50% of the minimum non-zero value observed for each respective protein, under the assumption that these missing values represented low-abundance proteins falling below the instrument's limit of detection. Finally, sample median normalization was applied across all samples to adjust for any latent variations in protein loading.

**1.1.2 Metabolomics Data Processing and Quality Control**

For metabolomics profiling, a pooled Quality Control (QC) sample was generated by combining equal volume aliquots from all experimental CSF samples. During instrumental data acquisition, the analytical sequence began with initial QC injections to equilibrate the LC-MS system, followed by the insertion of one QC sample after every five biological samples to monitor instrumental stability.

Following peak extraction via XCMS, a Quality Control-based robust LOESS signal correction (QC-RLSC) was mathematically fitted to the pooled QC data based on the explicit injection order to actively correct for temporal signal intensity drift. Subsequently, metabolic features exhibiting > 50% missingness in the pooled QC samples or > 80% missingness in the experimental biological samples were discarded. Missing values in the filtered matrix were then imputed utilizing the K-Nearest Neighbors (KNN) algorithm, followed by Probabilistic Quotient Normalization (PQN). As a final quality assurance step, features displaying a Coefficient of Variation (CV) > 50% within the pooled QC samples were strictly filtered out prior to downstream statistical analyses.

**1.2 Differential Analysis, Multivariate Modeling, and Functional Enrichment**

All bioinformatic workflows were executed in R (version 4.3.1). For the untargeted multi-omics profiling data, statistical significance between groups was assessed using Student's t-tests. Given the limited sample size (*n*=5 per group) in the discovery cohort, Benjamini-Hochberg false discovery rate (FDR) correction was excessively conservative, severely increasing the risk of Type II errors (false negatives). Therefore, nominal *p*-values (*p*<0.05) were utilized for the initial high-throughput screening to preserve biological signal discovery [1-3], and features identified under these nominal criteria are operationally defined as candidate differentially expressed proteins and metabolites (DEPs and DEMs) throughout the exploratory discovery phase. To strictly control for potential false positives arising from this screening approach, all downstream multi-omics leads were rigorously anchored by orthogonal quantitative assays in an independent cohort.

**1.2.1 Proteomics Differential and Functional Enrichment Analysis**

Differentially expressed proteins (DEPs) were defined using normative criteria: Fold Change (FC) > 1.5 or < 0.67 and a nominal *p*-value < 0.05. Functional enrichment of the identified DEPs was conducted using the clusterProfiler R package (version 4.10.0) by mapping the proteins to both the Gene Ontology (GO, release 2025-01-01) and KEGG databases (release 113.0). Pathways with a hypergeometric *p*-value < 0.05 were deemed significantly enriched. To further explore the topological architecture of the protein interactome, a protein-protein interaction (PPI) network was constructed utilizing the STRING database (version 12.0) and subsequently clustered via the Markov Cluster Algorithm (inflation parameter=1.5) to extract tightly connected functional modules.

**1.2.2 Metabolomics Multivariate Modeling, Differential, and Enrichment Analysis**

Following data preprocessing and normalization, the imported metabolite feature matrix was verified for identifier uniqueness. Any duplicated metabolite IDs were systematically resolved by appending unique suffixes (via the *make.unique* function in R) to ensure matrix integrity (remaining 486 unique features).

Due to the high-dimensional nature of metabolomic data, supervised multivariate discriminative modeling was conducted on this unified matrix using the ropls R package (version 1.38.0). Both Partial Least Squares-Discriminant Analysis (PLS-DA) and Orthogonal PLS-DA (OPLS-DA) models were constructed under identical parameters, including standard scaling and a uniform 100-permutation test. Recognizing the mathematical limitations of traditional *k*-fold cross-validation in small cohorts [4], both models were strictly evaluated using Leave-One-Out Cross-Validation (LOOCV), representing the optimal validation standard for small sample sizes. While the PLS-DA model captured global sample separation, the OPLS-DA model was further implemented to filter orthogonal noise. Consequently, Variable Importance in Projection (VIP) scores derived from the OPLS-DA model were utilized as the multivariate criterion for downstream feature selection.

Differentially expressed metabolites (DEMs) were subsequently defined using a multi-parameter criteria: FC > 1.5 or < 0.67, Student’s t-test *p*-value < 0.05, and OPLS-DA VIP > 1. Following feature selection, functional enrichment for the identified DEMs was specifically performed using the clusterProfiler R package (version 4.10.0) against the KEGG database (Note: Exogenous xenobiotics potentially introduced during surgery, such as anesthetic agents, lack human endogenous KEGG IDs and were thus algorithmically excluded from the functional pathway enrichment analysis.), with a hypergeometric *p*-value < 0.05 indicating significant pathway enrichment.

**1.3 Data Integration Analysis for Biomarker discovery using Latent cOmponents (DIABLO)**

Multi-omics integration was performed using the DIABLO framework (block sPLS-DA) via the mixOmics R package (version 6.30.0) to evaluate multi-omics group discrimination and explore highly contributive cross-omics features [5]. To focus the algorithm specifically on pathologically relevant variances, extract the most reliable cross-omics biological axes, and inherently mitigate the risk of high-dimensional overfitting, the model was strategically inputted with the rigorously pre-filtered significant molecules (216 DEPs and 26 DEMs).

Given the limited discovery cohort (*n*=10), model performance and component selection were evaluated using Leave-One-Out Cross-Validation (LOOCV). The cross-validation demonstrated reliable discriminative performance, with the metabolomics block achieving a 0% balanced error rate (BER) and the proteomics block achieving a 30% BER at the first component. To optimally capture orthogonal biological covariance for 2D topological visualization while conservatively preventing overfitting, a two-component model (ncomp=2) was established, and feature selection was strictly fixed to the top 10 discriminative features per omics layer (keepX = c(10, 10)).

**1.4 Targeted ELISA Validation for GOT2**

To quantify the absolute concentration of the candidate hub protein GOT2 in the cerebrospinal fluid (CSF), a commercial human GOT2 Sandwich-ELISA kit (Cusabio, China; Catalog No. CSB-E0960h) was utilized in strict accordance with the manufacturer’s protocol. Prior to the assay, CSF samples were thawed, centrifuged to remove any latent particulates, and assayed strictly undiluted in duplicate.

Briefly, 100 μL of standard solutions and biological samples were added to the designated microtiter wells and incubated for 2 hours at 37°C. Following complete liquid removal without washing, 100 μL of biotin-conjugated antibody was added per well and incubated for 1 hour at 37°C. The plate was then washed three times using the provided wash buffer. Subsequently, 100 μL of HRP-avidin was added and incubated for 1 hour at 37°C, followed by a stringent five-time washing step. For colorimetric development, 90 μL of TMB substrate was added and incubated in the dark at 37°C for 15-30 minutes, after which the reaction was terminated with 50 μL of Stop Solution. The optical density (OD) was immediately measured at 450 nm within 5 minutes using a microplate reader.

Absolute protein concentrations were calculated using Curve Expert software (version 1.3), where the standard curve was mathematically fitted using a rational function model selected for optimal goodness-of-fit (r>0.99). Notably, as this assay is fundamentally based on structural antigen-antibody binding, the final readouts (expressed in mU/mL) represent kit-specific concentration units reflecting physical protein mass, rather than functional catalytic enzyme activity.

**1.5 Targeted LC-MS/MS Validation of Metabolites**

To provide orthogonal validation and absolute quantification of the critical targeted metabolites, an ultra-performance liquid chromatography-tandem mass spectrometry (UHPLC-MS/MS) approach was performed. By strictly matching the chromatographic retention times and specific exact mass MS/MS transitions of the CSF samples with those of authentic chemical standards, this targeted assay definitively verified the metabolite identities, achieving the Metabolomics Standards Initiative (MSI) Level 1.

**1.5.1 Sample Preparation and Extraction**

Metabolite extraction was initiated by adding 180 μL of LC-MS grade methanol to 60 μL of CSF sample. The mixture was vortexed for 30 seconds and incubated at -20°C for 1 hour to ensure complete protein precipitation. Following centrifugation at 12,000 rpm for 15 minutes at 4°C, 80 μL of the resulting supernatant was carefully transferred to LC vials for direct instrumental analysis.

**1.5.2 UHPLC-MS/MS Conditions**

Chromatographic separation was performed on a Thermo Vanquish UHPLC system equipped with an Agilent ZORBAX Eclipse Plus C18 column (2.1 mm×150 mm, 1.8 μm). The column and auto-sampler temperatures were maintained at 35°C and 10°C, respectively. The mobile phases consisted of solvent A (0.1% formic acid and 10 mM ammonium formate in water) and solvent B (methanol). A 10-minute elution gradient was applied at a constant flow rate of 300 μL/min: 0–2.0 min, 10% B; 2.0–5.0 min, 10% to 90% B; 5.0–7.0 min, hold at 90% B; 7.1–10.0 min, return to 10% B for re-equilibration. The injection volume was 2 μL.

Mass spectrometric detection was executed using a Thermo TSQ Altis Plus triple quadrupole mass spectrometer in positive electrospray ionization (ESI+) mode. The source parameters were optimized as follows: capillary voltage, +3500 V; sheath gas, 30 Arb; auxiliary gas, 10 Arb; sweep gas, 0 Arb; ion transfer tube temperature, 325°C; and vaporizer temperature, 300°C. Data acquisition was performed in Multiple Reaction Monitoring (MRM) mode to ensure maximal sensitivity and selectivity.

**1.5.3 Calibration and Method Validation**

All data acquisition and quantitative calculations were processed using Xcalibur software (version 4.5). Absolute quantification was achieved by establishing calibration curves via stepwise dilution of authentic standard solutions. Linear regression with a 1/X weighting factor was applied, yielding high linearity (r > 0.999). According to the US FDA bioanalytical guidelines, limits of detection (LOD) and limits of quantitation (LOQ) were rigorously defined at signal-to-noise (S/N) ratios of 3 and 10, respectively. The method’s precision (Relative Standard Deviation [RSD] < 6%) and accuracy (average recovery > 95%) were strictly validated using Quality Control (QC) replicates, verifying the reliability of the targeted quantification.

**1.6 Targeted Clinical Covariate and Cross-Omics Correlation Analyses**

To rigorously contextualize the quantitative findings and address potential clinical confounders, targeted correlation analyses were performed exclusively within the independent TSCI validation cohort (*n*=8). The absolute concentrations of the validated molecules derived from the targeted assays were evaluated against key clinical covariates to serve distinct pathophysiological and technical purposes.

First, variables including the initial CSF laboratory indices were analyzed to systematically evaluate and rule out potential quantitative artifacts driven by intramedullary hemorrhage and blood-spinal cord barrier (BSCB) disruption. Second, time post-injury (duration) and AIS grade were evaluated to investigate the potential influence of clinical heterogeneity on the molecular signatures within the hyper-acute window. For this correlation analysis, baseline AIS grades were numerically coded (AIS A=1, AIS B=2). Furthermore, to independently assess the metabolic associations proposed by the multi-omics integration network, cross-omics correlations were calculated across the entire validation cohort (*n*=16) between the absolute quantitative levels of the candidate markers.

Given the utilization of raw absolute concentrations without normalization or logarithmic transformation, along with the non-normal distribution typical of clinical parameters in severe trauma, the non-parametric Spearman's rank correlation coefficient was strictly employed for all statistical assessments. The corresponding scatter plots and correlation coefficients (r) were generated to visualize these associations, with a *p*-value < 0.05 considered statistically significant.

**References**

1. Wang Z, Huang X, Tan H, Liang J, Li Z, Shen J. Proteomic Comparison of Paraspinal Muscle Imbalance Between Idiopathic Scoliosis and Congenital Scoliosis. Neurospine. 2023 Jun;20(2):709-724.
2. Yang B, Zhu Y, Li K, Wang F, Liu B, Zhou Q, et al. Machine learning model base on metabolomics and proteomics to predict cognitive impairment in Parkinson's disease. NPJ Parkinsons Dis. 2024 Oct 11;10(1):187.
3. Abudurexiti M, Abuduhalike R, Naman T, Wupuer N, Duan D, Keranmu M, et al. Integrated proteomic and metabolomic profiling reveals novel insights on the inflammation and immune response in HFpEF. BMC Genomics. 2024 Jul 8;25(1):676.
4. Vabalas A, Gowen E, Poliakoff E, Casson AJ. Machine learning algorithm validation with a limited sample size. PLoS One. 2019 Nov 7;14(11):e0224365.
5. Singh A, Shannon CP, Gautier B, Rohart F, Vacher M, Tebbutt SJ, Lê Cao KA. DIABLO: an integrative approach for identifying key molecular drivers from multi-omics assays. Bioinformatics. 2019 Sep 1;35(17):3055-3062.
